# Diversity in Infection Specificity between the Bloom-forming Microalga *Heterosigma akashiwo* and Its dsDNA Virus, Heterosigma akashiwo Virus

**DOI:** 10.1264/jsme2.ME23036

**Published:** 2023-06-09

**Authors:** Yusaku Funaoka, Haruna Hiromoto, Daichi Morimoto, Michiko Takahashi, Kei Wada, Keizo Nagasaki

**Affiliations:** 1 Faculty of Agriculture and Marine Science, Kochi University, Nankoku, Kochi 783–8502, Japan; 2 Faculty of Science and Technology, Kochi University, Nankoku, Kochi 783–8502, Japan; 3 Kochi Medical School, Kochi University, Nankoku, Kochi 783–8505, Japan; 4 Department of Medical Sciences, University of Miyazaki, Miyazaki 889–1692, Japan

**Keywords:** Heterosigma akashiwo virus, most probable number assay, red tide, infection specificity, cross-infectivity test

## Abstract

Heterosigma akashiwo virus (HaV) is a dsDNA virus that infects the bloom-forming raphidoflagellate *Heterosigma akashiwo*. Both the host and its virus are phenotypically diverse in terms of infection specificity. Their relationships have been examined based on the occurrence or absence of algal lysis following virus inoculation; however, variations in the strain-level host-virus relationship regarding infectivity and lysis rates remain unclear. Therefore, we performed a series of cross-infectivity tests using 60 *H. akashiwo* and 22 HaV strains isolated from the coastal waters of western Japan. The host strains were divided into 5 different groups and viruses into 4 groups. Using a representative strain from each group, algal lysis was observed in 14 of the (5×4=) 20 host-virus combinations; the concentration of infectious units in each HaV suspension was then assessed using the most probable number (MPN) assay on the five host strains. Virus titers ranged between 1.1×10^1^ and 2.1×10^7^ infectious units mL^–1^; the titer of each viral lysate was differently estimated using distinct *H. akashiwo* strains as hosts. These results suggest that (1) a clonal viral lysate comprises virions with different intraspecific infection specificities and/or (2) the efficiency and error rates of each intracellular replication process vary in each host-virus combination.

*Heterosigma akashiwo* (Hada) Hada ex Hara & Chihara is a noxious bloom-forming raphidophyte that is distributed worldwide (Honjo, 1993; Gómez *et al.*, 2022). It forms red tides that often cause the mortality of farmed fish, such as salmon (Chang *et al.*, 1990). Therefore, a more detailed understanding of the ecological factors involved in the disintegration of *H. akashiwo* blooms is imperative for coastal management (Imai *et al.*, 1993; Khan *et al.*, 1997; Martínez *et al.*, 2010; Kim *et al.*, 2015; Sandoval-Sanhueza *et al.*, 2022).

Viral infections are an important factor regulating the abundance of phytoplankton (Brussaard, 2005). Virus-like particles in *H. akashiwo* cells were initially discovered almost three decades ago in samples collected at the final stage of a red tide event (Nagasaki *et al.*, 1994a, 1994b). Viral titers were shown to significantly increase with a decline in the abundance of *H. akashiwo* (Tarutani *et al.*, 2000). These findings strongly suggest that viruses regulate bloom dynamics and contribute to the disintegration of red tides (Nagasaki *et al.*, 1994a, 1994b; Tarutani *et al.*, 2000).

Several RNA and DNA viruses that infect *H. akashiwo* have been isolated to date and include Heterosigma akashiwo RNA virus (HaRNAV), Heterosigma akashiwo nuclear inclusion virus (HaNIV), OIs1 (unregistered double-stranded DNA virus infecting *H. akashiwo*), and Heterosigma akashiwo virus (HaV) (Nagasaki and Yamaguchi, 1997; Lawrence *et al.*, 2001, 2006; Tai *et al.*, 2003). Among them, HaV is the most intensively investigated virus for its intraspecific infection specificity (Nagasaki and Yamaguchi, 1998). Even *H. akashiwo* and HaV strains simultaneously isolated from the same sampling sites showed highly diverse intraspecific infectivity (Tarutani *et al.*, 2000; Tomaru *et al.*, 2004). This finding also suggests that viral infections are one of the factors affecting not only the cell density, but also the strain-level diversity of the *H. akashiwo* population.

Intrastrain interactions between *H. akashiwo* and HaV have been examined based on a qualitative evaluation, *i.e.*, the presence/absence of algal lysis after a virus inoculation (Nagasaki and Yamaguchi, 1998; Tarutani *et al.*, 2000; Tomaru *et al.*, 2004). However, it currently remains unclear whether the lysis event for each host and virus combination is a result of similar extracellular and intracellular processes, including infection programs and host responses. The interactions between the phage infection program and host antiviral systems in infected bacterial cells are well documented (Labrie *et al.*, 2010). Similarly, the bloom-forming eukaryotic microalga *Emiliania huxleyi* has been shown to defend‍ ‍itself against viral infection by a metabolic shift (Rosenwasser *et al.*, 2014) or transitioning from the diploid to haploid phase (Frada *et al.*, 2008). These interactions may affect not only the success (or failure) of infection, but also the efficiency of viral infection and productivity of progeny virions. However, these parameters have been overlooked by conventional qualitative assessments (Tarutani *et al.*, 2000; Tomaru *et al.*, 2004).

In the present study, we quantitatively assessed the infection and lysis of HaV using cross-infectivity tests, and proposed plausible hypotheses to explain the marked variations observed in the viral titers of each host strain.

## Materials and Methods

### Sampling

Water samples were taken from the surface water layer with a bucket and from above the sediment-water interface using a RIGO-B transparent water bottle (Rigosha). Samples were collected once or twice a week between 26 March and 26 July 2021 in‍ ‍Uranouchi Inlet, Kochi Prefecture, Japan (33°24′31.7″N, 133°21′31.8″E). In this area, *H. akashiwo* blooms occur annually between April and July (Fig. S1 and S2). Sampling was performed between 9:30 and 10:30 AM to match circadian rhythms in the vertical migration of this taxa (Nagasaki *et al.*, 1996). Water samples were transported to the laboratory in a cooler box, and *H. akashiwo* cells were counted under an optical microscope (CKX53SF; OLYMPUS). To increase accuracy, counting was conducted after vigorously vortexing an aliquot of a seawater sample to immobilize swimming *H. akashiwo* cells.

### Isolation of *H. akashiwo* and virus strains

*H. akashiwo* cells were randomly isolated on the same day using the micropipette method (Tomaru *et al.*, 2004). Briefly, a single *H. akashiwo* cell was picked up from seawater samples and washed several times in sterile seawater. The dilution-to-extinction method was used to isolate *H. akashiwo* cells in samples with a high population density. All strains were cultured in Daigo’s IMK medium (Nihon Pharmaceutical) at 22°C under a 14-h/10-h light/dark photocycle (light intensity: 345‍ ‍μmol photons m^–2^ s^–1^). The taxonomic characterization of the isolated strains was performed using a microscopy ana­lysis and the partial sequences of 18S rRNA genes (Coyne *et al.*, 2005). DNA was extracted using a modified xanthogenate-SDS (XS) method (Tillett and Neilan, 2000). Briefly, 1‍ ‍mL of the *H. akashiwo* culture was centrifuged at 855×*g* for‍ ‍5‍ ‍min, followed by the addition of 550‍ ‍μL of freshly prepared XS buffer (8‍ ‍mg of potassium ethyl xanthogenate; 120‍ ‍mM Tris-HCl, pH 7.4; 30‍ ‍mM EDTA, pH 8; 1% sodium dodecyl sulfate; 408‍ ‍μL of sterile water). Samples were incubated at 70°C for 60‍ ‍min and then placed on ice for 30‍ ‍min. Cell debris was removed by centrifugation at 21,400×*g* for 15‍ ‍min, and the supernatant was‍ ‍carefully transferred to new tubes. An equal volume of phenol:chloroform:isoamyl alcohol (25:24:1) was added and the tube was mixed several times by inversion. Samples were then centrifuged at 21,400×*g* for 10‍ ‍min, and the same process was‍ ‍repeated. The resultant aqueous phase was extracted with chloroform:isoamyl alcohol (24:1). DNA was precipitated by isopropanol, washed with 70% ethanol, and dissolved in 20‍ ‍μL of TE buffer. PCR was performed in a 25-μL reaction mixture containing 1‍ ‍μL of extracted DNA and 0.8‍ ‍μM each of the Hs 1350F and Hs 1705R primers (Coyne *et al.*, 2005). The amplicon size was estimated to be 376 bp. Thermal cycling conditions were as follows: denaturation at 98°C for 10‍ ‍s, primer annealing at 60°C for 30‍ ‍s, and an extension at 72°C for 1‍ ‍min. PCR products were subjected to Sanger sequencing.

Seawater samples for the isolation of HaV were filtered through polycarbonate membrane filters with a pore size of 0.2‍ ‍μm (Whatman plc, Cytiva). Filtrates (300‍ ‍μL) were inoculated into *H. akashiwo* cultures (800‍ ‍μL) and incubated under the conditions described above. The host strains used for virus isolation are shown in Table S1. The dilution-to-extinction method with serial 10-fold dilutions was used for virus isolation. Viral lysates were diluted with sterile seawater. Serial 10-fold dilutions (100‍ ‍μL) were inoculated with exponentially growing cultures of *H. akashiwo* (150‍ ‍μL) in 96-well plates (Thermo Fisher Scientific) and incubated until lysis occurred (7–10 days). The clonal isolation of HaV was achieved after two cycles of the dilution-to-extinction method using lysates from the most diluted wells (Nagasaki and Yamaguchi, 1997).

Novel HaV isolates were identified using HaV-specific PCR. Genomic DNA was extracted using the phenol/chloroform method (Saunders, 1993). Briefly, 1‍ ‍mL of the viral lysate was mixed with an equal volume of phenol:chloroform:isoamyl alcohol (25:24:1). The aqueous phase was separated by centrifugation at 21,400×*g* for 5‍ ‍min and transferred to a new tube. DNA was then extracted by adding an equal volume of chloroform and then precipitated with isopropanol. DNA was washed with 70% ethanol and dissolved in TE buffer. PCR amplification was performed in 25‍ ‍μL of a PCR mixture containing 1‍ ‍μL of HaV-DNA and 0.4‍ ‍μM each of the PAGB01A and PAGB01B primers, targeting a 425-bp region of the putative ATPase gene of previously isolated HaV01 (Nagasaki *et al.*, 2001). Amplification was performed under the following thermal cycling conditions: 30 cycles of amplification with denaturation at 98°C for 10‍ ‍s, primer annealing at 54°C for 30‍ ‍s, and an extension reaction at 72°C for 1‍ ‍min. PCR products were then subjected to Sanger sequencing in order to identify HaV (Nagasaki *et al.*, 2001).

### Cross-infectivity test

Viral isolates were propagated by repeatedly infecting the original host strain used for isolation (Table S1). In cross-infectivity tests, freshly produced viral lysates were centrifuged at 855×*g* at 4°C for 5‍ ‍min to remove cell debris. An aliquot of the lysate (10‍ ‍μL) was inoculated with 150‍ ‍μL of the host culture in 96-well plates and incubated under the same conditions described above for 10–14 days. Each well was monitored daily using a microscope. The results of the assays were subdivided into three categories: i) “Complete lysis”, characterized by the almost complete disappearance of host cells at the end of the incubation period, ii) “Incomplete lysis”, characterized by a temporal decline in host cells during the first few days of the incubation and subsequent recovery towards the end of the incubation, and iii) “No lysis”, defined as all other cases.

### Enumeration of total viral particles

To assess the replication of viral particles, 10‍ ‍μL of the viral lysate was added to 1‍ ‍mL of the host culture and incubated for 4 days under the above-described conditions. After 4 days, subsamples from both the inoculated lysate and infected cultures were taken and collected on a 0.02-μm filter (WHA68096002; Whatman plc, Cytiva). The filters were washed with sterile water, dried, and subsequently stained with SYBR Gold (Thermo Fisher Scientific), which was conducted with a slight modification to the method reported by Patel *et al.* (2007). The number of viral particles was measured using epifluorescence microscopy (BX60F5; OLYMPUS) equipped with the FITC filter set (excitation 495‍ ‍nm; emission 537‍ ‍nm).

### Quantification of infectious viruses

The titration of viral lysates against representative host strains was performed by the most probable number (MPN) assay to estimate the concentration of infectious viruses (Suttle and Chan, 1993). *H. akashiwo* strain U13262, which was sensitive to all the representative strains, was used to prepare representative viral lysates. A 10-fold serial dilution of each viral lysate (100‍ ‍μL) was inoculated with 150‍ ‍μL of the selected *H. akashiwo* cultures growing at the early to mid-log phase (2.0×10^4^‍ ‍cells‍ ‍mL^–1^) and was then incubated for two weeks under the same conditions as those described above. The concentration of infectious units in each viral lysate was enumerated using the MPN calculator (MPNcalc v1.2.0).

### Statistical ana­lysis

Hierarchical clustering using the Euclidean distance and average linkage metrics was used to analyze data from cross-infectivity tests with the R package “stats” (version 4.2.1). Gap statistics were calculated by “clusGap” in the “cluster” package (version 2.1.3) to measure the optimal number of clusters (Tibshirani *et al.*, 2001). The significance of differences (*P*<0.01, *n*=3) in increases in viral particles was assessed by the Tukey-Kramer test using the “multcomp” (version 1.4-20) package in R (Hothorn *et al.*, 2008). The same statistical tests were applied to detect significant differences in MPN values between each host-virus combination.

### Data availability

The nucleotide sequences of the PCR amplicons of *H. akashiwo* strains U13262 and U14275 as well as HaV120 were deposited in the DDBJ/EMBL/GenBank database under accession numbers LC752797, LC752798, and LC752947, respectively.

## Results

### Establishment of clonal cultures of *H. akashiwo* and HaV

During the sampling period, *H. akashiwo* cell density initially peaked on 15 April (3.5×10^4^‍ ‍cells‍ ‍mL^–1^) and then decreased on 6 May (33 cells mL^–1^; Fig. S2). It increased again on 11 May (2.4×10^3^‍ ‍cells‍ ‍mL^–1^) and then rapidly collapsed by 18 May (Fig. S2). The second *H. akashiwo* bloom occurred on 7 July (Fig. S2). *H. akashiwo* cell density at the bottom layer was similar to that of surface samples, except for that on 31 March (Fig. S2). During the sampling period, we successfully isolated 46 algal and 21 viral strains from Uranouchi Inlet using the micropipette and dilution-to-extinction methods (Table S1). PCR fragments from the algal 18S rRNA gene (376 bp) and the putative viral ATPase gene (425 bps) were amplified from 44 out of the 46 host strains and all 21 virus strains, respectively. Two algal strains (U13269 and U14154) were taxonomically identified based only on morphological features because they were lost after the cross-infectivity test. The sequence identities of host- and virus-PCR products were 99.65–100% (to the *H. akashiwo* gene for 18S ribosomal RNA; LC214009.1) and 100% (to a putative ATPase gene of HaV; AB028864.1), respectively. Therefore, all of the algal and viral strains isolated in the present study were identified as *H. akashiwo* and HaV.

### Diversity in host susceptibility and viral infectivity

**Fig. 1.**
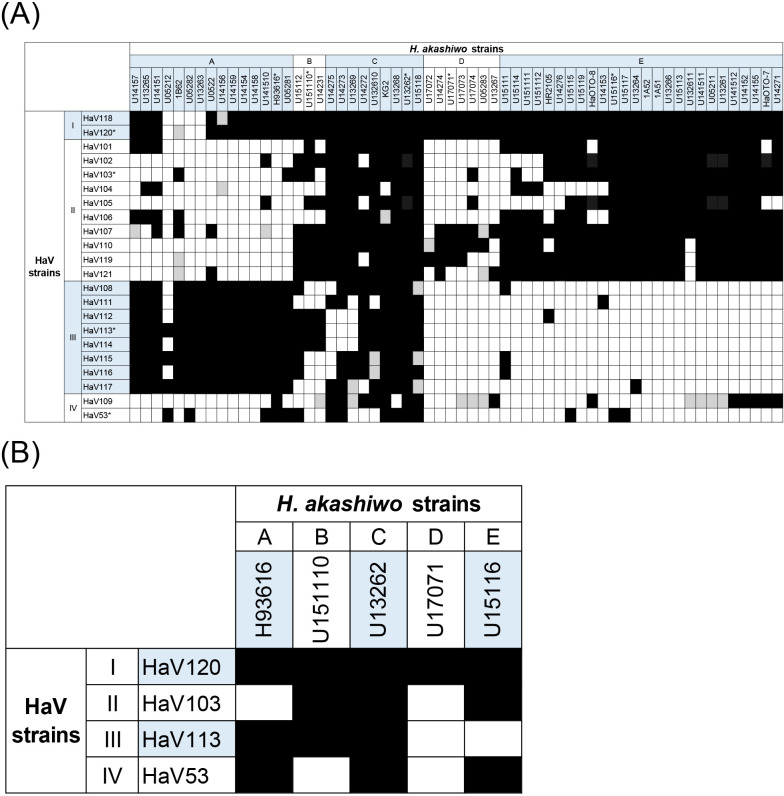
Viral susceptibility of *Heterosigma akashiwo* strains to HaV strains isolated from western Japan. (A) HaV and *H. akashiwo* strains were classified into 4 (I, II, III, and IV) groups and 5 (A, B, C, D, and E) groups, respectively, based on infection specificity. Black, gray, and white colors indicate complete lysis, ambiguous lysis (*e.g.*, restoration of host growth), and all other cases, respectively, as described in the Methods section. (B) Representative strains (*) from each viral group and host group were selected for further ana­lyses.

We performed cross-infectivity tests using 60 host and 22 viral strains, including some provided by other institutions (Table S1), to characterize viral infectivity and host susceptibility. Based on gap statistics of the susceptibility patterns of *H. akashiwo*, all of the tested strains were divided into five groups (A, B, C, D, and E; Fig. 1A, S3, and S4). HaV strains were subdivided into three groups based on their infectivity patterns (Fig. S3 and S4). We then used differences between susceptibility and infection patterns to further subdivide viral strains into four representative groups (Fig. 1A and S4). For example, HaV strains assigned to Group I lysed most of the *H. akashiwo* strains isolated from Uranouchi Inlet (93–95%; Fig. 1A). Viral strains in Group II caused lysis in more than 50% of *H. akashiwo* strains (50–82%; Fig. 1A). Group III viruses were infectious to only approximately 40% of *H. akashiwo* strains (37–42%; Fig. 1A). The previously isolated virus HaV53 (Group IV; isolated from Hiroshima Bay) infected only 25% of the *H. akashiwo* strains isolated from Uranouchi Inlet (Fig. 1A). In addition, the inoculation of HaV109 in the same group showed ambiguous results, such as the restoration of host growth (also observed in other viral groups; Fig. 1A). Therefore, although all strains originated from western Japan, viral infectivity and host susceptibility were highly diverse, which was consistent with previous studies (Tarutani *et al.*, 2000; Tomaru *et al.*, 2004). Considerable diversity in infection specificity was observed even among strains isolated from the same sampling station. We then selected a representative strain from each host group and virus group for further ana­lyses in subsequent experiments (Fig. 1B).

### Viral multiplication

To assess the impact of viral infection, we measured the abundance of viral particles by SYBR Gold staining. Cell lysis in ‘infectious’ host-virus combinations (*e.g.*, HaV120 vs. U13262) was accompanied by a marked increase in the number of viral particles (Fig. S5). However, viral particle numbers did not show a marked increase in the ‘less infectious combination’ (*e.g.*, HaV120 vs. U17071; Fig. S5).

### MPN assay using five *H. akashiwo* clones as the host

**Fig. 2.**
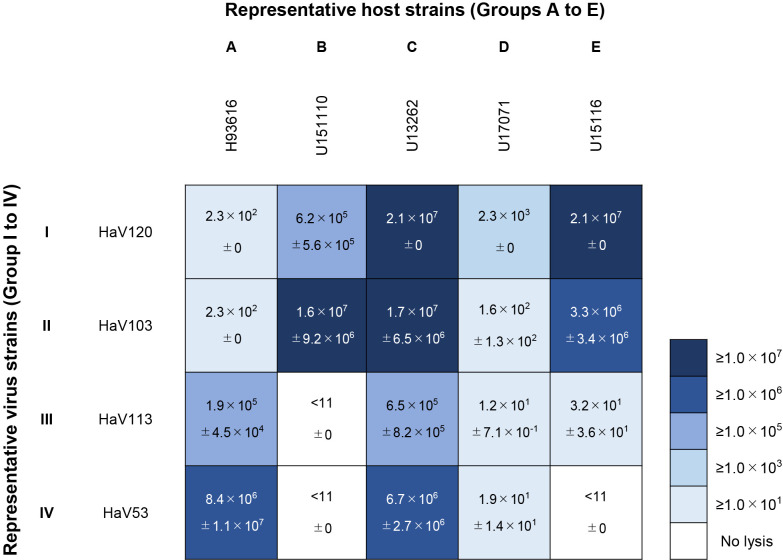
Results of titration between representative *Heterosigma akashiwo* and HaV strains shown in Fig. 1B. The numbers in each cell represent the concentration of infectious units (infectious units mL^–1^). Values show the average and standard deviation (*n*=3). The color gradient (lower right corner) represents low (light blue) and high (dark blue) titers.

The infectivities of the four representative viral strains against the five representative host strains were estimated by the MPN assay (Fig. 2), which showed high variability among the tested host-virus combinations (Fig. S6). For example, the abundance of the HaV120 isolate (Group I) varied between 2.3×10^2^ and 2.1×10^7^ infectious units mL^–1^, depending on the host infected (Fig. 2 and S6). Similarly, other HaV isolates showed marked variability in the concentration of infectious units between the five host strains used in the assays (Fig. 2 and S6). Unexpected or no lysis occurred in seven out of the 20 host-virus combinations, contradicting the results of the cross-infectivity tests (see above; Fig. 1B and 2). For example, the MPN assay showed that HaV103 produced 2.3×10^2^ and 1.6×10^2^ infectious units mL^–1^ when inoculated with *H. akashiwo* strains H93616 and U17071, respectively, whereas no virulence was observed in cross-infectivity tests. Furthermore, viral strains HaV113 and HaV53 lysing *H. akashiwo* strains U151110 and U15116, respectively, were below the detection limit (<11 infectious units mL^–1^; Fig. 2) when assessed by MPN.

## Discussion

In the present study, we established culture collections of *H. akashiwo* and HaV strains, which appeared to be monospecific based on the partial gene sequences of 18S rRNA and a putative ATPase, respectively. Similar to previous field surveys in Hiroshima Bay (Tarutani *et al.*, 2000; Tomaru *et al.*, 2004), host and virus strains both exhibited variable susceptibility and infectivity in respect to each other (Fig. 1). This result suggests that various types of *H. akashiwo* and HaV strains coexist in Uranouchi Inlet.

The present study builds upon existing literature by demonstrating variations in the titers of infectious viruses, which depend on the combination of a virus and host strain, thereby offering insights into the relationship between the algal host and its virus. Even though viruses in the lysates were clonal, their titers markedly varied among the host strains used in MPN assays, ranging between 10^1^ and 10^7^ infectious units mL^–1^ (Fig. 2). Since various types of hosts and viruses coexist in the aquatic environment, their interrelationships are considered to be more complex than previously considered.

In some host-virus combinations, contradicting findings were observed between cross-infectivity tests and MPN assays (Fig. 1 and 2). Assuming that the only difference between these two assays was the host strain used to prepare viral lysates, we speculated that host-dependent post-translational modifications in viral proteins affect their virulence. However, further studies are needed to elucidate the mechanisms underlying this phenomenon.

**Fig. 3.**
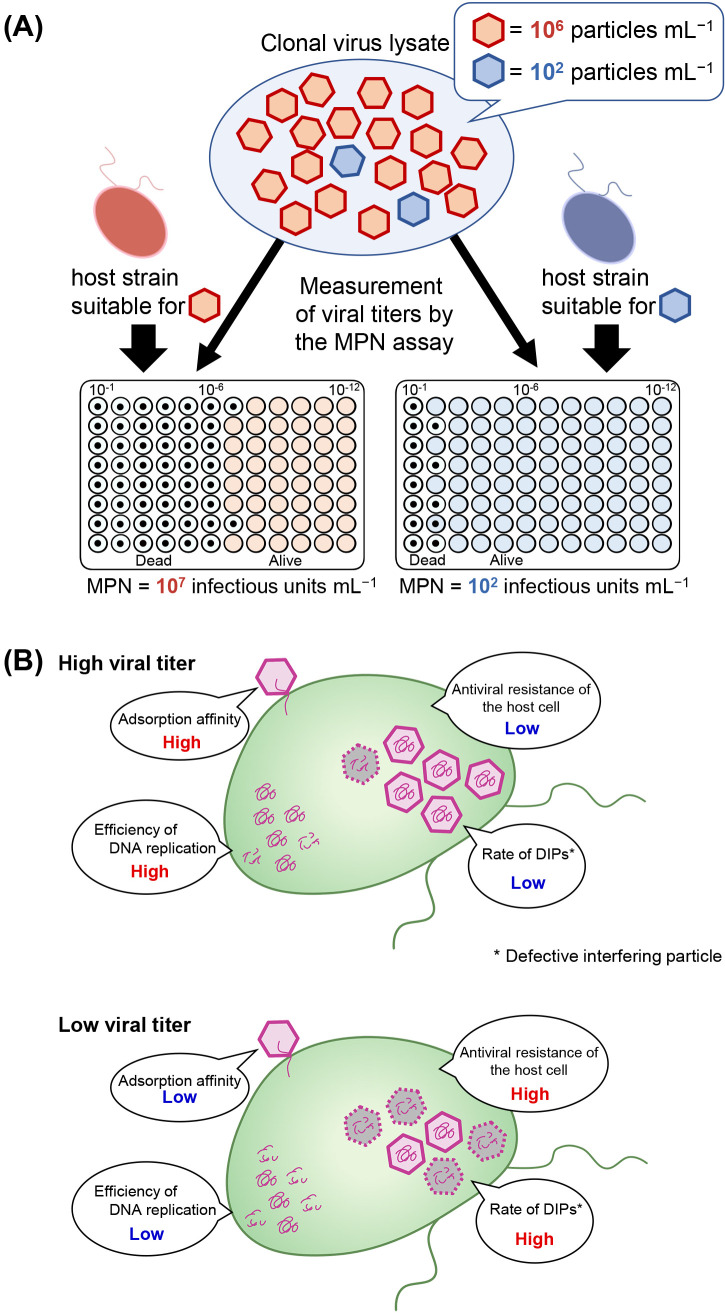
Possible mechanisms for viruses exhibiting high variability in titers depending on host strains. (A) A clonal HaV lysate may be comprised of multiple types of virions, which differ in terms of their infection specificities. The viral progeny in a single host strain lysate is genetically identical, but has diverse phenotypic infection specificities. The resultant MPN of the lysate is assessed by the susceptibility of the host strain used for titration. (B) Impact of efficiency and error rates of the intracellular replication process on the production of progeny viruses in a viral infected cell. Host-virus combinations with high replication efficiency and low error rates result in a high virus titer, while host-virus combinations with low replication efficiency, the activation of host responses, and the generation of defective particles result in a low virus titer.

Differences in the titration of clonal HaV lysates using distinct *H. akashiwo* strains as hosts remain unclear. One hypothesis is that virions in the clonal viral lysate show different intraspecies host specificities (Fig. 3A). In this scenario, if a large percentage of progeny virions exhibit infectivity to a specific host strain, its titer needs to be evaluated as high as long as the same host strain is used in the MPN assay and *vice versa* (Fig. 3A).

The mechanisms responsible for the diversity of virions have yet to be clarified. By using the combination of MPN and DAPI (4′,6-diamidino-2-phenylindole) staining, Tarutani *et al.* (2006) demonstrated that only a small percentage (3–4%) of HaV virions were infective to a *H. akashiwo* strain. These findings indicate that the remaining 96–97% of virions are not considered to be infectious to the host strain used in the MPN assay. Therefore, the infection specificity of virions may be heterogeneous at any rate even though the viral lysate is clonal.

The efficiency and/or error rate of each intracellular multiplication process may vary in each host-virus combination (Fig. 3B). This may affect the percentage of defective viral particles in the progeny, thereby leading to a decreased titer of infectious viruses. Tarutani *et al.* (2006) indicated that viral adsorption on the host cell surface was essential for HaV to initiate its infection; therefore, the low adsorption affinity of some viruses may indirectly result in their lower titers than those of viruses with high adsorption affinity. As‍ ‍shown for viruses infecting cyanobacteria, cellular responses to viral infections differ between host strains (Doron *et al.*, 2016) and at various stages of infection (Zborowsky and Lindell, 2019). Collectively, these findings suggest that the efficiency and error rates of DNA replication and virion morphogenesis affect viral titers in each specific combination of *H. akashiwo* and HaV strains. However, intracellular interactions between HaV and infected cells remain unclear (Fig. 3B). In addition, we discovered that some *H. akashiwo* strains regained growth after marked decreases in cell numbers caused by HaV infection (Fig. 1), suggesting that unknown mechanisms confer antiviral resistance to *H. akashiwo* cells.

In conclusion, the present study revealed the interesting phenomenon of clonal HaV viruses exhibiting high variability in titers depending on the host strain infected. The underlying mole­cular mechanisms have yet to be elucidated. Furthermore, this phenomenon may not be unique to this species. Therefore, the relationships between algae and their‍ ‍viruses reported in previous studies may need to be reviewed. The host and viral clonal cultures established in‍ ‍the present study are expected to facilitate our understanding of the complex interactions between the red tide-causing alga *H. akashiwo* and HaV.

## Citation

Funaoka, Y., Hiromoto, H., Morimoto, D., Takahashi, M., Wada, K., and Nagasaki, K. (2023) Diversity in Infection Specificity between the Bloom-forming Microalga *Heterosigma akashiwo* and Its dsDNA Virus, Heterosigma akashiwo Virus. *Microbes Environ ***38**: ME23036.

https://doi.org/10.1264/jsme2.ME23036

## Supplementary Material

Supplementary Material
