# Supplementary material for: Diversity in Infection Specificity between the Bloom-forming Microalga Heterosigma akashiwo and Its dsDNA Virus, Heterosigma akashiwo Virus
Source: Microbes Environ. 2023 Jun 9;38(2):ME23036. doi: 10.1264/jsme2.ME23036 (PMC10308240; doi:10.1264/jsme2.ME23036)
Supplement: Supplementary file 1 — Supplementary Material [file 38_23036_s1.pdf]

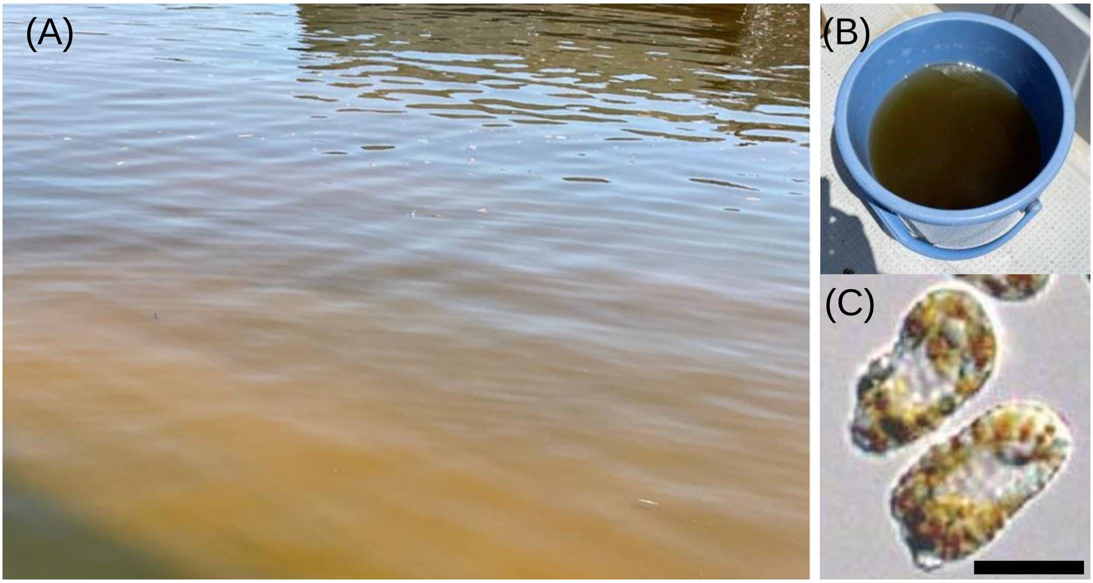

Fig. S1. *Heterosigma akashiwo* bloom. (A) Image of algal community dominated by *H. akashiwo* in Uranouchi Inlet, Kochi, Japan. (B) Water sample collected from water surface. (C) Optical micrograph of *H. akashiwo* cells. Scale bar indicates 10 μm.

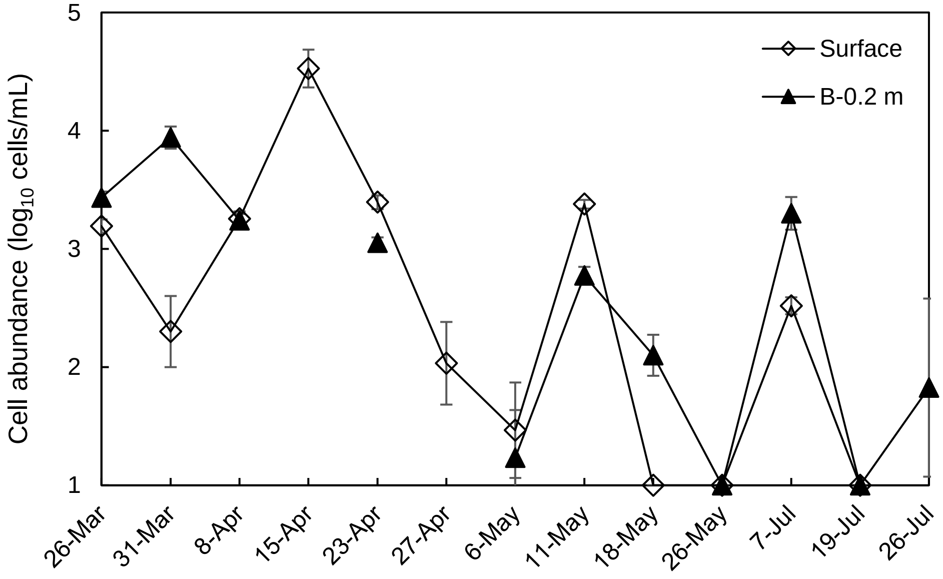

Fig. S2. Changes in cell abundance of *Heterosigma akashiwo* in Uranouchi Inlet, Kochi, Japan, from late March through late July 2021. White diamonds and black triangles indicate cell abundance in surface layer and just (0.2 m) above the bottom layer, respectively.

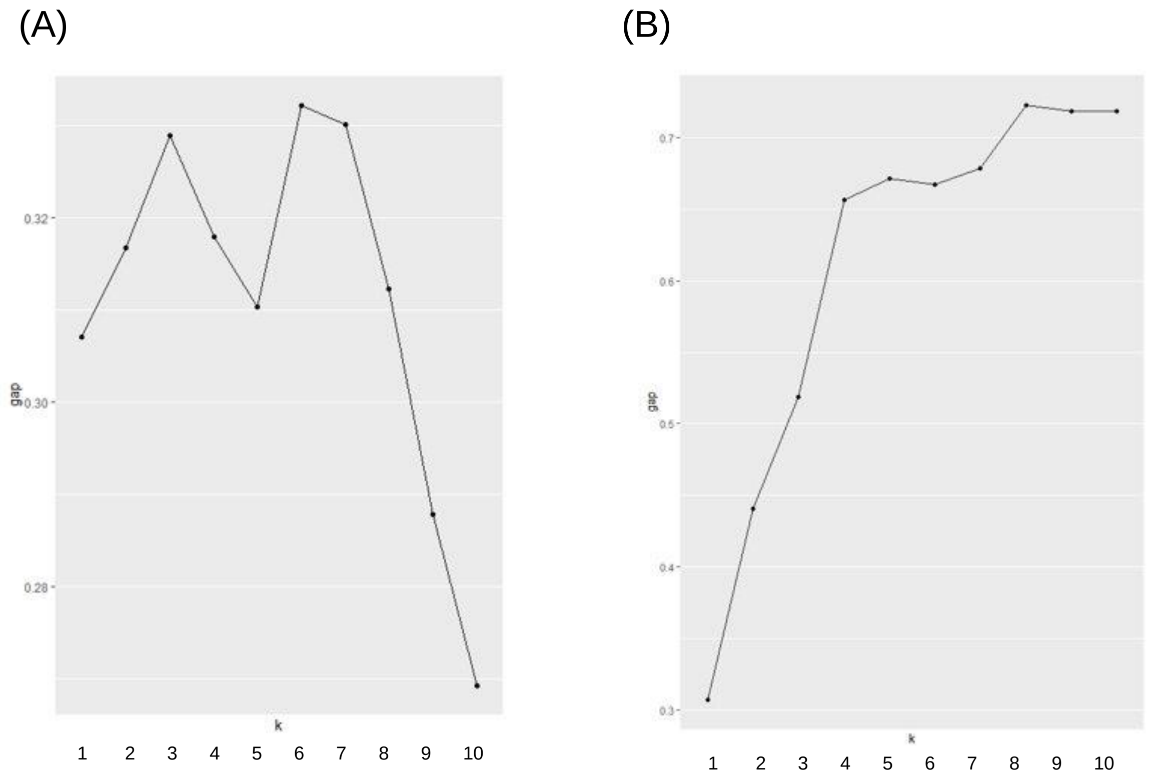

Fig. S3. Results of gap statistics used to estimate the optimal number of clusters for virus- (A) and host- strains (B).

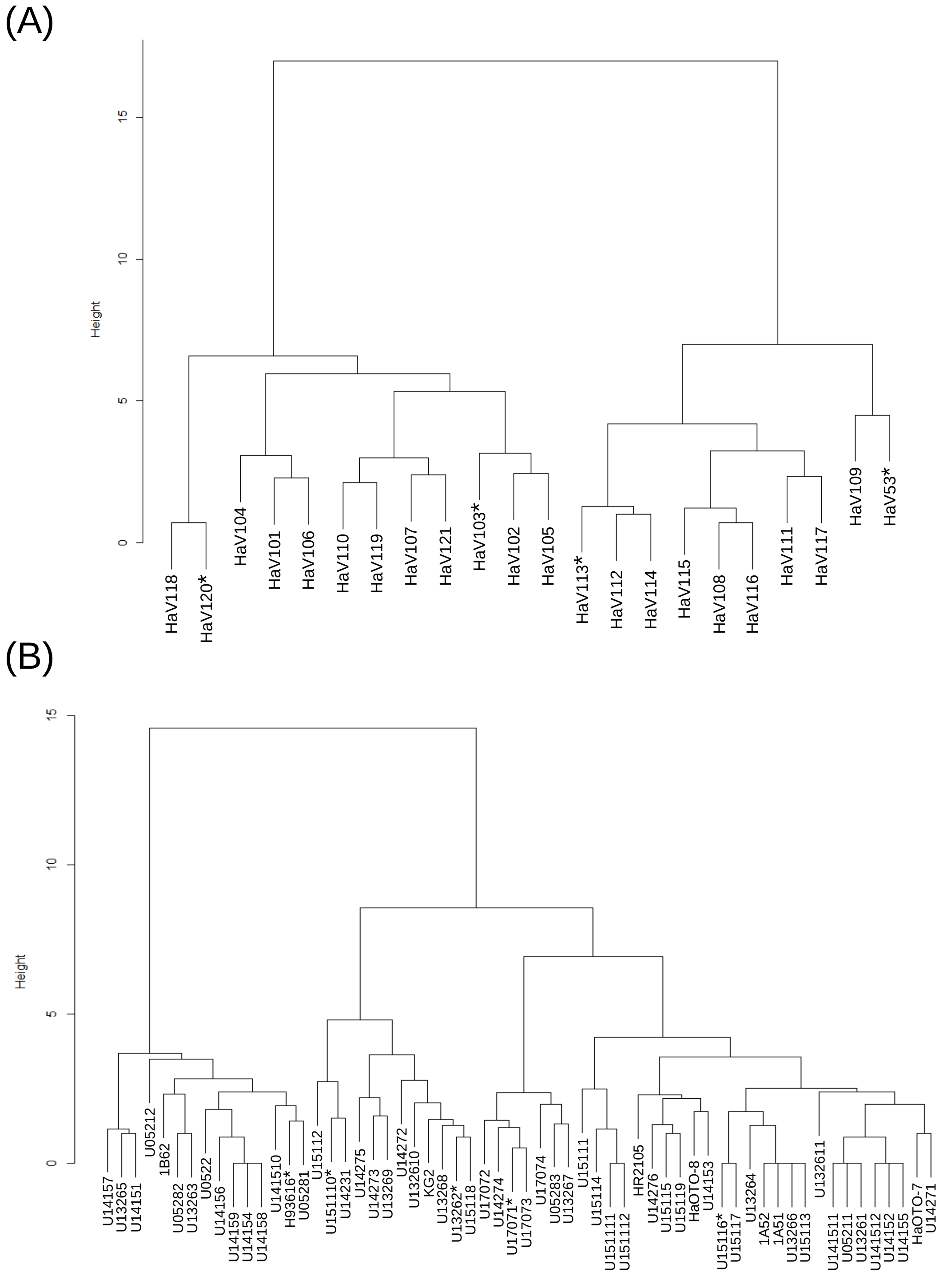

Fig. S4. Hierarchical clustering of virus- (A) and host-strains (B) based on viral infectivity and host susceptibility patterns. The optical cluster number was estimated by gap statistics analysis (Fig. S3); asterisks (*) indicate representative strains in each group.

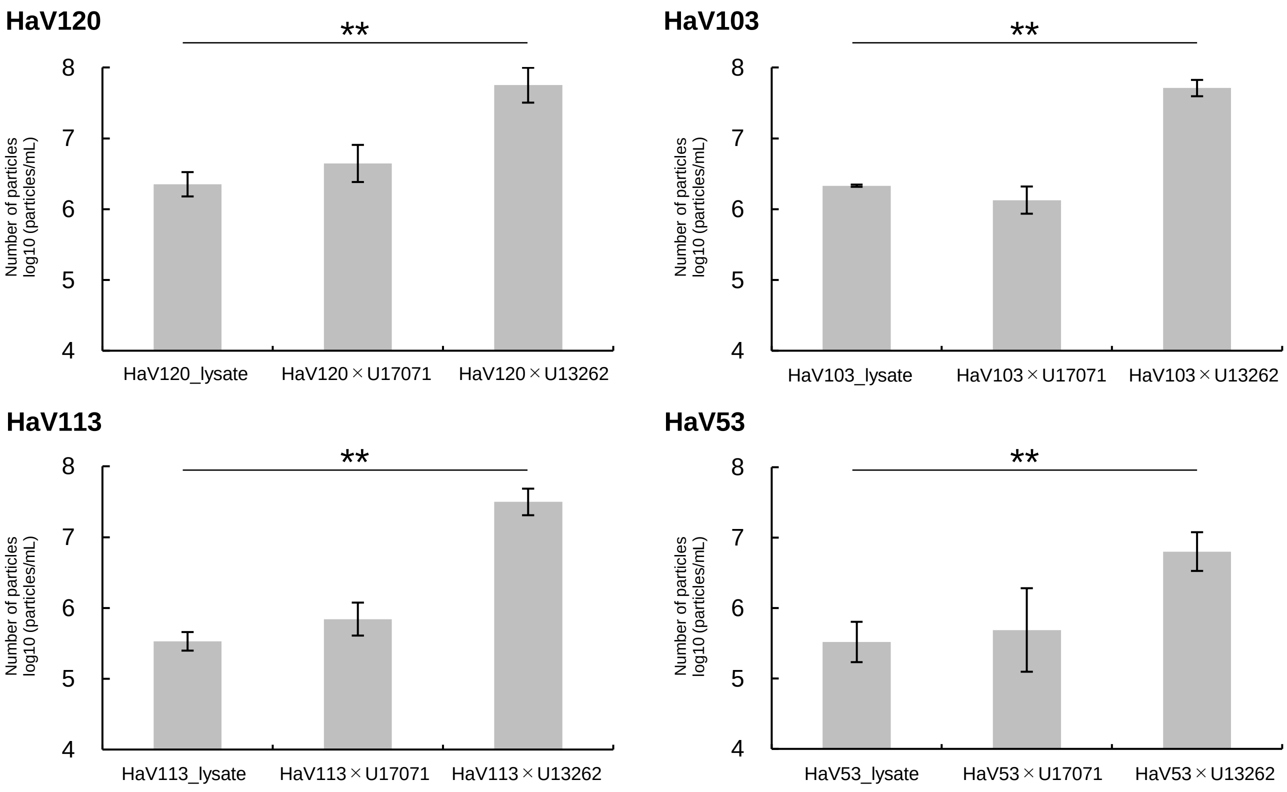

Fig. S5. Impacts of infection of the representative HaV strains on virus production in highly susceptible and less susceptible host strains (*Heterosigma akashiwo* U13262 and U17071, respectively; refer Fig. 2). Original viral lysates were prepared with *H. akashiwo* U13262 as host. Values indicate average and standard deviation; asterisks indicate statistical significance (**$p < 0.01$).

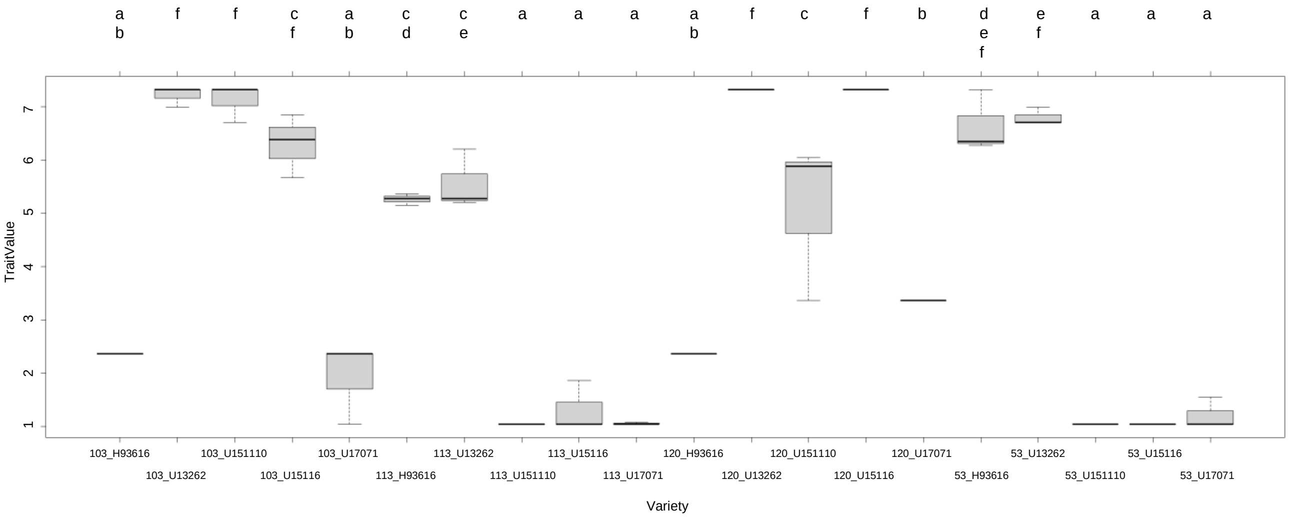

Fig. S6. Boxplots to support the Tukey-Kramer pairwise comparisons of viral titers shown in Fig. 2; solid black bar within each box represents median; upper alphabets mean summarized results of Tukey-Kramer test; letters denote significant differences in mean titers with group a < b < c < d < e < f.

Table S1. Lists of *Heterosigma akashiwo* and HaV strains used in this study. (A) *H. akashiwo* strains, (B) HaV strains.

(A) *H. akashiwo* strains

Strain name	Location	Date	Provided by
U13261	Uranouchi Inlet, Kochi, Japan	2021-03-26	-
U13262*	Uranouchi Inlet, Kochi, Japan	2021-03-26	-
U13263	Uranouchi Inlet, Kochi, Japan	2021-03-26	-
U13264	Uranouchi Inlet, Kochi, Japan	2021-03-26	-
U13265	Uranouchi Inlet, Kochi, Japan	2021-03-26	-
U13266	Uranouchi Inlet, Kochi, Japan	2021-03-26	-
U13267	Uranouchi Inlet, Kochi, Japan	2021-03-26	-
U13268	Uranouchi Inlet, Kochi, Japan	2021-03-26	-
U13269	Uranouchi Inlet, Kochi, Japan	2021-03-26	-
U132610	Uranouchi Inlet, Kochi, Japan	2021-03-26	-
U132611	Uranouchi Inlet, Kochi, Japan	2021-03-26	-
U14151	Uranouchi Inlet, Kochi, Japan	2021-04-15	-
U14152	Uranouchi Inlet, Kochi, Japan	2021-04-15	-
U14153	Uranouchi Inlet, Kochi, Japan	2021-04-15	-
U14154	Uranouchi Inlet, Kochi, Japan	2021-04-15	-
U14155	Uranouchi Inlet, Kochi, Japan	2021-04-15	-
U14156	Uranouchi Inlet, Kochi, Japan	2021-04-15	-
U14157	Uranouchi Inlet, Kochi, Japan	2021-04-15	-
U14158	Uranouchi Inlet, Kochi, Japan	2021-04-15	-
U14159	Uranouchi Inlet, Kochi, Japan	2021-04-15	-
U141510	Uranouchi Inlet, Kochi, Japan	2021-04-15	-
U141511	Uranouchi Inlet, Kochi, Japan	2021-04-15	-
U141512	Uranouchi Inlet, Kochi, Japan	2021-04-15	-
U14231	Uranouchi Inlet, Kochi, Japan	2021-04-23	-
U14271	Uranouchi Inlet, Kochi, Japan	2021-04-27	-
U14272	Uranouchi Inlet, Kochi, Japan	2021-04-27	-
U14273	Uranouchi Inlet, Kochi, Japan	2021-04-27	-
U14274	Uranouchi Inlet, Kochi, Japan	2021-04-27	-
U14275	Uranouchi Inlet, Kochi, Japan	2021-04-27	-
U14276	Uranouchi Inlet, Kochi, Japan	2021-04-27	-
U15111	Uranouchi Inlet, Kochi, Japan	2021-05-11	-
U15112	Uranouchi Inlet, Kochi, Japan	2021-05-11	-
U15113	Uranouchi Inlet, Kochi, Japan	2021-05-11	-
U15114	Uranouchi Inlet, Kochi, Japan	2021-05-11	-
U15115	Uranouchi Inlet, Kochi, Japan	2021-05-11	-
U15116	Uranouchi Inlet, Kochi, Japan	2021-05-11	-
U15117	Uranouchi Inlet, Kochi, Japan	2021-05-11	-
U15118	Uranouchi Inlet, Kochi, Japan	2021-05-11	-
U15119	Uranouchi Inlet, Kochi, Japan	2021-05-11	-
U151110	Uranouchi Inlet, Kochi, Japan	2021-05-11	-
U151111	Uranouchi Inlet, Kochi, Japan	2021-05-11	-
U151112	Uranouchi Inlet, Kochi, Japan	2021-05-11	-
U17071	Uranouchi Inlet, Kochi, Japan	2021-07-07	-
U17072	Uranouchi Inlet, Kochi, Japan	2021-07-08	-
U17073	Uranouchi Inlet, Kochi, Japan	2021-07-09	-
U17074	Uranouchi Inlet, Kochi, Japan	2021-07-10	-
U05211	Uranouchi Inlet, Kochi, Japan	2020-05-21	-
U05212	Uranouchi Inlet, Kochi, Japan	2020-05-21	-
U0522	Uranouchi Inlet, Kochi, Japan	2020-05-22	-
U05281	Uranouchi Inlet, Kochi, Japan	2020-05-28	-
U05282	Uranouchi Inlet, Kochi, Japan	2020-05-28	-
U05283	Uranouchi Inlet, Kochi, Japan	2020-05-28	-
HaOTO-7	Uranouchi Inlet, Kochi, Japan	2019-5	-
HaOTO-8	Uranouchi Inlet, Kochi, Japan	2019-5	-
HR2105	Bingo-Nada, Hiroshima, Japan	2021-5	Fisheries Research and Education Agency, Japan
1A51	Urasoko Inlet, Kagoshima, Japan	2015-06-03	Kagoshima University, Japan
1A52	Urasoko Inlet, Kagoshima, Japan	2015-06-03	Kagoshima University, Japan
1B62	Urasoko Inlet, Kagoshima, Japan	2015-06-10	Kagoshima University, Japan
KG2	Kagoshima Inlet, Kagoshima, Japan	2021-03-11	Kagoshima University, Japan
H93616	Hiroshima Bay, Hiroshima, Japan	1993-6-16	Fisheries Research and Education Agency, Japan

* used to prepare lysate for MPN assay and EFM

(B) HaV strains

Strain name	Location	Date	Host strain used for virus isolation
HaV101	Uranouchi Inlet, Kochi, Japan	2021-04-08	U05211
HaV102	Uranouchi Inlet, Kochi, Japan	2021-04-14	U05211
HaV103	Uranouchi Inlet, Kochi, Japan	2021-04-14	HaOTO-8
HaV104	Uranouchi Inlet, Kochi, Japan	2021-04-15	U05211
HaV105	Uranouchi Inlet, Kochi, Japan	2021-04-15	U05211
HaV106	Uranouchi Inlet, Kochi, Japan	2021-04-15	U05211
HaV107	Uranouchi Inlet, Kochi, Japan	2021-04-15	U05211
HaV108	Uranouchi Inlet, Kochi, Japan	2021-04-15	U0522
HaV109	Uranouchi Inlet, Kochi, Japan	2021-04-15	U05283
HaV110	Uranouchi Inlet, Kochi, Japan	2021-04-15	HaOTO-8
HaV111	Uranouchi Inlet, Kochi, Japan	2021-04-15	U05282
HaV112	Uranouchi Inlet, Kochi, Japan	2021-04-23	U05282
HaV113	Uranouchi Inlet, Kochi, Japan	2021-04-23	U05282
HaV114	Uranouchi Inlet, Kochi, Japan	2021-04-23	U05282
HaV115	Uranouchi Inlet, Kochi, Japan	2021-04-23	U0522
HaV116	Uranouchi Inlet, Kochi, Japan	2021-04-23	U0522
HaV117	Uranouchi Inlet, Kochi, Japan	2021-04-23	U0522
HaV118	Uranouchi Inlet, Kochi, Japan	2021-05-06	HaOTO-8
HaV119	Uranouchi Inlet, Kochi, Japan	2021-05-11	HaOTO-8
HaV120	Uranouchi Inlet, Kochi, Japan	2021-05-18	HaOTO-8
HaV121	Uranouchi Inlet, Kochi, Japan	2021-05-26	HaOTO-7
HaV53	Hiroshima Bay, Hiroshima, Japan	1998-07-01	H93616
